# Chain‐End Grafting of RAFT Polyelectrolyte Macroligands to Stabilize Near‐Infrared‐Emitting Gold Nanoclusters

**DOI:** 10.1002/marc.202600007

**Published:** 2026-04-09

**Authors:** Bárbara Casteleiro, Pierre Alcouffe, Fernande Da Cruz, Agnès Crépet, José Manuel Gaspar Martinho, Marie‐Thérèse Charreyre, José Paulo Sequeira Farinha, Arnaud Favier

**Affiliations:** ^1^ Centro De Química Estrutural Institute of Molecular Sciences and Department of Chemical Engineering Instituto Superior Técnico University of Lisbon Lisbon Portugal; ^2^ Univ Lyon Laboratoire Ingénierie des Matériaux Polymères Université Lyon 1, INSA de Lyon, CNRS, UMR5223 Villeurbanne France

**Keywords:** gold nanoclusters, near‐infrared photoluminescence, polyelectrolytes, RAFT polymerization

## Abstract

With their photoluminescence properties, gold nanoclusters (AuNCs, d<2 nm) show great promise in various fields, including sensing, imaging and photosensitization. However, important challenges remain both regarding their colloidal stability and surface functionalization. Here, we investigate the synthesis of AuNCs with end‐grafted polymer chains bearing multiple functional lateral groups. Water‐soluble polymer chains are synthesized by RAFT controlled radical polymerization to present positive or negative lateral charges, and a thiol end‐group to promote grafting to gold. These polyelectrolyte chains are obtained by copolymerization of *N*‐acryloylmorpholine with a comonomer, either cationic (acryloylamino propyltrimethylammonium chloride, AAPTA) or anionic (2‐acrylamido‐2‐methyl‐1‐propanesulfonate, AMPS), at different charge densities. The AuNCs are synthesized in situ in the presence of the macromolecular ligands, directly in water at room temperature. The cationic copolymers successfully lead to highly stable, NIR‐emitting AuNCs fully characterized in terms of size (HR‐TEM, ^1^H DOSY NMR) and spectrophysical properties. The anionic copolymers yield instead a mixture of AuNCs and larger gold nanoparticles. This study offers an important insight into the influence of electrostatic charges and functional groups of the polymer ligand on the stabilization and optical physical properties of the AuNCs. In addition, it paves the way for the preparation of a large range of functionalized AuNCs.

## Graphical abstract

Surface‐functionalized gold nanoclusters (AuNCs) are synthesized in situ by chain‐end grafting of RAFT polyelectrolytes at room temperature directly in water. It leads to highly stable polymer‐AuNCs nanohybrids emitting near‐infrared photoluminescence with µs lifetimes.

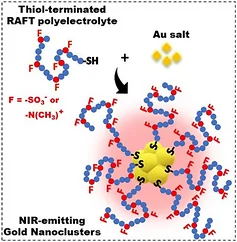

## 1 Introduction

Gold Nanoclusters (AuNCs) are gold nanostructures with less than 2–3 nm in size (200 to 300 Au atoms), exhibiting great promises in material science. In the 1970s, the first examples of AuNCs were stabilized in solution using different phosphines [1], before the Brust group demonstrated in 1994 the potential of using small thiol molecules as alternative ligands [2]. The method was quickly adopted and optimized, for example by the group of Wheten et al., that demonstrated a better control over the AuNCs growth [3]. Since then, a growing number of synthetic routes and stabilization methods have been developed, increasing the application potential of AuNCs in various fields such as catalysis, bioimaging, and sensing [4, 5, 6, 7, 8].

The importance of the molecular ligand at the surface of the AuNCs has been widely investigated as it is known to influence their colloidal stability and optical properties, impacting their possible applications [1, 9, 10]. The most commonly used ligands are small thiol molecules like mercaptohexanoic acid (MHA) [9], even though the resulting AuNCs generally exhibit low photoluminescence quantum yield (c.a. 0.1%) and low colloidal stability [11, 12]. However, other ligands, such as the tri‐peptide glutathione (GSH), allow the preparation of small AuNCs with higher stability, owing to multiple carboxyl and amino coordination sites besides thiol, and the good encapsulation provided by intermolecular hydrogen bonding and electrostatic interactions [9]. Such ligands have also been associated with higher photoluminescence quantum yield of the charge transfer band [13], due to the encapsulation and the strong charge transfer interactions provided by the electron‐rich atoms and groups able to transfer electrons to the Au kernel [1, 6]. Alternatively, larger biomolecules such as proteins [14, 15, 16, 17] and linear polymer chains have also recently been used as protecting ligands [18, 19, 20, 21, 22]. Their shielding effect provides better resistance to external factors, such as changes of temperature, pH, and ionic strength.

We have previously demonstrated the successful synthesis of “hairy” AuNCs stabilized by thiol‐terminated polymer chains produced by RAFT controlled radical polymerization. Water‐soluble and biocompatible poly(*N*‐acryloyl morpholine) (PNAM‐SH) chains with different molecular weights (ranging from 3 000 to 11 000 g mol^−1^) were used as macro‐ligand to prepare, directly into water, polymer‐protected AuNCs (PNAM‐AuNCs), with significantly improved colloidal stability (> 2 months) [22]. These AuNCs exhibit NIR photoluminescence (λ_em max_ = 710 nm) with an enhanced quantum yield (5% to 12%, depending on the molecular weight of the PNAM‐SH chain) and longer emission lifetimes (1 to 2 µs) than equivalent AuNCs prepared with small thiol ligands like MHA‐AuNCs (quantum yields <0.1% and lifetimes around 0.5 µs).

This original approach opens up a new avenue to produce highly stable NIR‐emitting AuNCs with tunable surface properties. For instance, we showed that polystyrene chain‐end‐stabilized AuNCs can be prepared for their subsequent encapsulation in polystyrene‐*block*‐polyethyleneglycol micelles [23]. In addition, such an approach can be used to functionalize the surface of the AuNCs. Indeed, RAFT polymerization not only provides excellent control over the molecular weight and low size dispersity of the polymer chains, but it readily provides a thiol ω‐chain‐end for anchoring to gold nanomaterials [22, 24]. Furthermore, RAFT offers huge versatility regarding the composition of the polymer [25], and can also be used to introduce different functional groups on the polymer, either in lateral or α‐end positions of the chains [24, 25, 26].

To demonstrate this strategy, here we explored the synthesis of AuNCs, directly in the presence of copolymer chains presenting different types and densities of electrostatic charges in lateral groups, in addition to a thiol ω‐end‐group to promote an oriented grafting to the gold surface (Figure 1).

**FIGURE 1:**
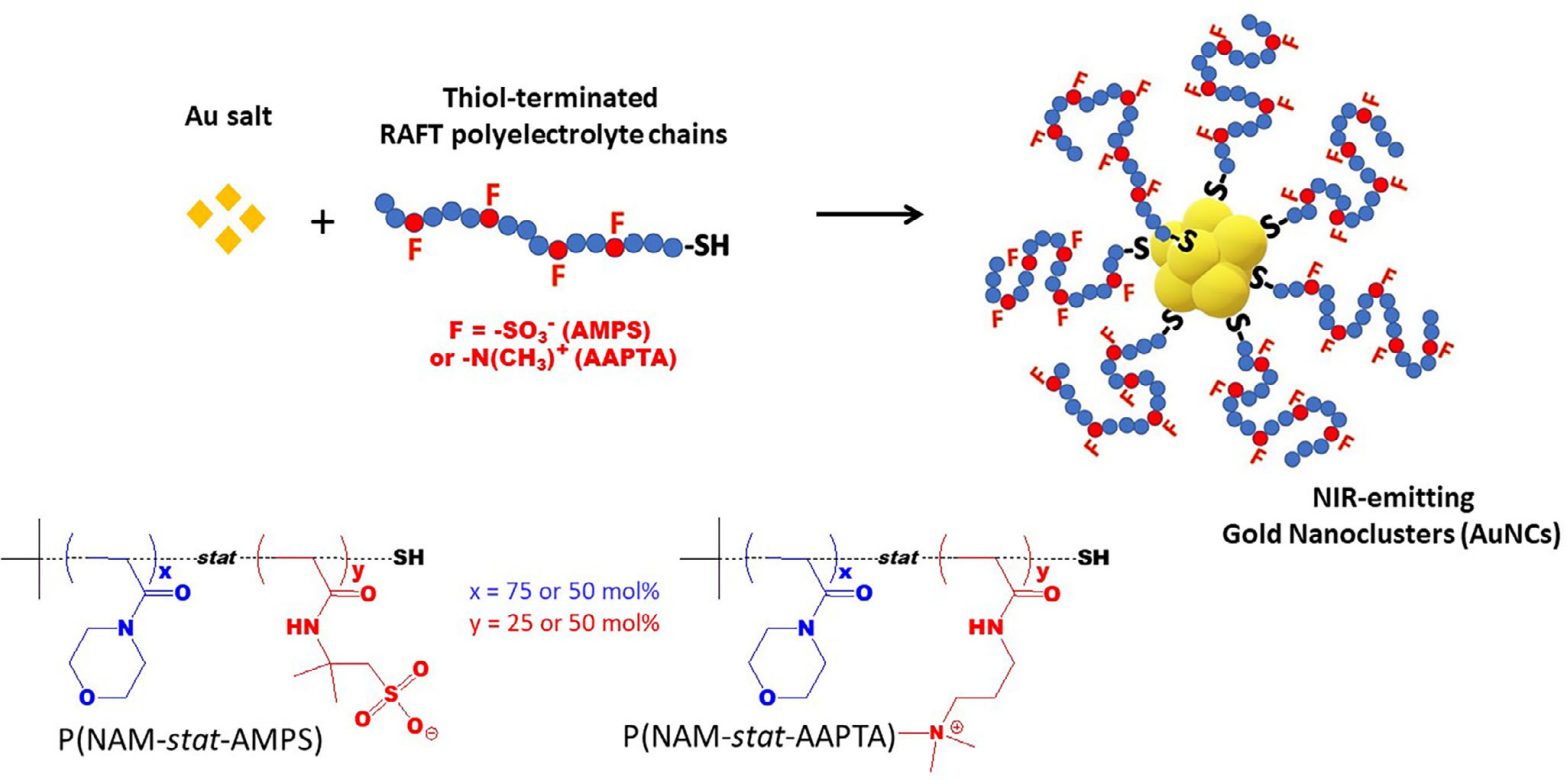
Schematic representation of the synthesis of AuNCs stabilized by thiol‐terminated polyelectrolyte chains of cationic poly(*N*‐acryloylmorpholine‐*stat*‐acryloylaminopropyl trimethyl ammonium chloride) P(NAM‐*stat*‐AAPTA) or anionic poly(*N*‐acryloylmorpholine‐*stat*‐2‐acrylamido‐2‐methyl‐1‐propanesulfonic acid) P(NAM‐*stat*‐AMPS) obtained by RAFT copolymerization.

Charged ligands are known to improve the dispersion and stability of gold nanoparticles in aqueous media through electrostatic interactions. In addition, they may be used to produce various kinds of ordered structures, with peptides or proteins, by self‐assembly via electrostatic complexation [27, 28]. Ligands containing a sulfonate or a quaternary amine group have the advantage of being charged over a large pH range, unlike carboxylate or less substituted amines. Moreover, sulfonate or quaternary amine groups can interact with gold themselves, providing further stabilization of the nanostructures [29] (even though the gold‐thiol interaction is stronger). Thiol small molecular ligands bearing a sulfonate or a quaternary amine group have already been considered to stabilize various types of gold nanostructures, but rarely AuNCs. For example, 3‐mercapto‐1‐propanesulfonate and p‐mercaptobenzenesulfonic acid (p‐MBSA) sulfonated molecular thiol ligands have been used to prepare larger gold nanoparticles (AuNPs, diameter d > 2 nm), that have been considered for use as antiviral drugs for Dengue [30], or in catalysis [31]. However, very few examples were reported for the synthesis of smaller gold nanostructures like AuNCs. Recently, AuNCs produced with p‐MBSA have shown an enhanced stability to pH, ionic strength and temperature, compared to AuNCs obtained with a carboxylated ligands of similar size [32]. Molecular thiol ligands bearing a quaternary ammonium group, in particular 11‐trimethylammonium‐undecanethiol, were also used to stabilize AuNCs [33, 34, 35, 36], but the preparation of the clusters proved to be challenging due to the interaction between the positively charged ligand and the negatively charged Au(I) gold salt precursor [36].

The synthesis of AuNCs in situ in the presence of macromolecular ligands presenting multiple charges could provide even more stable AuNCs and would further combine the advantages of AuNCs with those of polyelectrolytes, especially in aqueous conditions. Polyelectrolytes are generally very water‐soluble and can be used to develop various kinds of assemblies via electrostatic interactions, for applications in the healthcare field. However, the synthesis of polyelectrolyte‐stabilized AuNCs is not straightforward since the charges can interfere with the nanocluster formation process that involves the reduction of gold salts in aqueous conditions [36]. Most examples reported in the literature refer to polymer‐gold assemblies (similarly to layer‐by‐layer approaches) obtained by combining polyelectrolytes without thiol groups, and gold nanostructures pre‐synthesized separately with small molecular ligands [37, 38, 39]. Very few studies have considered the synthesis of gold nanostructures directly in the presence of a polyelectrolyte and these have mainly involved the preparation of gold nanoparticles (AuNPs, d > 2 nm) with polycations without thiol anchoring groups [40, 41]. AuNCs have been produced in the presence of linear polyethylene imine (PEI), a well‐known cationic polymer used for gene delivery [42]. In the absence of thiol, the AuNCs were stabilized by multiple weak interactions with the PEI amines, probably leading to large polymer‐AuNCs assemblies. We did not find similar approaches in the presence of polyanions.

In this study, we investigated the possibility of using RAFT macro‐ligands (exhibiting a thiol end‐group) that are either polyanionic or polycationic, for the synthesis of stable AuNCs (Figure 1). The macro‐ligands were linear polymer chains obtained by RAFT random copolymerization of *N*‐acryloylmorpholine (NAM) with a charged comonomer, either (i) cationic (AAPTA, acryloylaminopropyltrimethylammonium chloride, bearing a permanent quaternary ammonium positive charge) or (ii) anionic (AMPS, 2‐acrylamido‐2‐methyl‐1‐propanesulfonic acid, bearing a permanent sulfonic acid negative charge). To better understand the influence of the polyelectrolyte charges, copolymers with varying charge densities were synthesized for the in situ preparation of AuNCs (in similar conditions to PNAM‐AuNCs) [22], and their size and photophysical properties were compared.

## 2 Experimental Section

### 2.1 Materials

Au(III) chloride trihydrate (HAuCl_4_.3H_2_O, ≥99.9%, Sigma‐Aldrich), sodium hydroxide (NaOH, pure pellets, Carlo Erba), sodium borohydride (NaBH_4_, >98.5%, Sigma‐Aldrich), Tris(2‐carboxyéthyl)phosphine hydrochloride (TCEP‐HCL, Pierce, Thermo Scientific), (3‐Acrylamidopropyl) trimethylammonium chloride (AAPTA, 75 wt.% H_2_O solution, Sigma‐Aldrich), and 1,3,5‐trioxane (≥99%, Sigma‐Aldrich) were used as received. *N*‐acryloylmorpholine (NAM, 97%, Sigma‐Aldrich) was distilled at 120°C and 10 mmHg. 1,4‐dioxane (≥99%, Sigma‐Aldrich) was distilled with LiAlH_4_ at 110°C and stored under argon at 4°C. 2,2’‐azobis(isobutyronitrile) (AIBN, Fluka) was recrystallized in ethanol. 2‐Acrylamido‐2‐methyl‐1‐propanesulfonic acid (AMPS, 99%, Aldrich) was purified by recrystallization in ethanol. Deionized water used for synthesis was generated by a Millipore Milli‐Q system (Millipore—Merck).

### 2.2 Polymer Synthesis

Thiol‐terminated poly(*N*‐acryloyl morpholine) (PNAM‐SH) homopolymers were synthesized according to a previously described procedure [43]. RAFT polymerization using *tert*‐butyl dithiobenzoate (tBDB) as chain transfer agent (CTA) was followed by aminolysis of the ω‐chain‐end in the presence of aminoethyl morpholine [43]. Thiol‐terminated copolymers (cationic poly(*N*‐acryloylmorpholine‐*stat*‐acryloylaminopropyl trimethyl ammonium chloride) P(NAM‐*stat*‐AAPTA) or anionic poly(*N*‐acryloylmorpholine‐*stat*‐2‐acrylamido‐2‐methyl‐1‐propanesulfonic acid) P(NAM‐*stat*‐AMPS)) were synthesized according to a previously described procedure [22], using tBDB as CTA. The aminolysis of the ω‐chain‐end for the charged copolymers was performed in aqueous medium using TCEP‐HCL (0.14 eq.), as for PNAM‐SH. The copolymers were purified by ultrafiltration with molecular weight cut‐off (MWCO) of 3 000 g mol^−1^ (regenerated cellulose, Amicon Ultra 4 mL, Millipore), before freeze‐drying.

### 2.3 AuNCs Synthesis

#### 2.3.1 PNAM‐AuNCs

The PNAM‐AuNCs were synthesized as previously described [22]. An aqueous solution of PNAM‐SH (12.9 mL, 1.88 mM, 24.3 µmol, 1.96 eq.) was added to a HAuCl_4_ aqueous solution (498 µL, 24.9 mM, 12.4 µmol, 1 eq.) stirred in a water bath set at 27°C. 1.088 M NaOH (508 µL, 0.55 mmol, 44.6 eq.) was added, followed dropwise by a freshly prepared NaBH_4_ solution in 0.2 M NaOH (201 µL, 0.1 M, 1.73 eq.). The mixture was stirred at 27°C for another 2 h, and the final brown dispersion was stored at room temperature.

#### 2.3.2 P(NAM‐*stat*‐AAPTA) and P(NAM‐*stat*‐AMPS) Stabilized AuNCs

The synthesis of AuNCs in the presence of P(NAM‐*stat*‐AAPTA)‐SH and P(NAM‐*stat*‐AMPS)‐SH was performed in similar conditions, except for the addition of a NaBH_4_ solution (1.73 eq. of NaBH_4_ added dropwise). The final mixture was stirred for 3 h at 27°C. The same protocol was followed for 25% AAPTA and 50% AAPTA copolymers.

### 2.4 Characterization Techniques

#### 2.4.1 Nuclear Magnetic Resonance (NMR)

The ^1^H NMR and ^1^H DOSY NMR experiments were recorded using a Bruker AVANCE III Spectrometer operating at 400 MHz with a 5 mm Z‐gradients BBFO+ probe [44]. For DOSY, the AuNCs samples were prepared at ca. 40 mg. mL^−1^ by solvent exchange to D_2_O by ultrafiltration with a 3 kDa molecular weight cut‐off (MWCO) regenerated cellulose filters (Amicon, Millipore). The spectra were recorded at 300K.

DOSY experiments in D_2_O were acquired using the Bruker LEDBPGP2S [45] pulse program (stimulated echo sequence with bipolar gradient pulses). The gradient pulse length δ was optimized to each sample and varied between 1000 and 5000 µs. The diffusion delay was set to 200 ms. For all experiments, gradients were linearly sampled from 0.978 to 47.921 G cm^−1^ in 40 points and the gradient pulses were smooth‐square shaped (SMSQ10.100). Sixty‐four scans were acquired on 16k data points (1 s recycle time). Fourier transform was applied to the free induction decays (FIDs) with 2 Hz Lorentzian line‐broadening using the Topspin software (Bruker) with automatic baseline correction. For each gradient strength value, the experimental signal amplitude (I) of an NMR resonance is given by the Stejskal‐Tanner (Equation 1) [46].

(1) $$I=I_{0}\;e^{-D\gamma^{2}g^{2}\delta^{2}\left(\Delta -\frac{\delta }{3}-\frac{\tau }{2}\right)}$$

where I_0_ is the reference amplitude (at zero gradient), D is the self‐ diffusion coefficient, ϒ is the gyromagnetic ratio of the observed nucleus, τ is the time between the bipolar gradient pulses, g is the gradient strength, δ is the gradient pulse length, and Δ is the diffusion time. The multi‐exponential fitting of the Stejskal‐Tanner curves were processed using Dynamic Center software (Bruker) in such a way to extract the average value of the diffusion coefficient of the nanoclusters. 2D ^1^H DOSY NMR maps were also obtained by a multiexponential fitting using the TopSpin software (Bruker).

The hydrodynamic diameter (*D*_h_) of the compounds was calculated by the Stokes‐Einstein (Equation 2) where D is the diffusion coefficient (m^2^.s^−1^), k_B_ the Boltzmann's constant, *T* the absolute temperature and η the viscosity (assuming that it is the same as the solvent) [22].

(2) $$D=\frac{k_{B}T}{3\pi \eta D_{h}}$$

#### 2.4.2 UV–vis Absorption and Photoluminescence

UV‐vis absorption spectra were recorded on an UV‐660 UV‐VIS Spectrophotometer from JASCO International (Tokyo, Japan) with a double monochromator and photomultiplier detector for high resolution, using a Peltier temperature controller. Luminescence emission spectra were recorded at right‐angle geometry on a Horiba‐Jobin Yvon Fluorolog 3–22 spectrofluorimeter equipped with a xenon lamp of 450 V. The software used was FluorEssence. The samples were analyzed at room ambient temperature, using 0.5 cm optical path length quartz cell. The fluorescence quantum yields were determined by the reference method using a Coumarin 102 solution in absolute ethanol (ϕ = 0.75, λ_exc_ = 354 nm; 400 nm< λ_em_ < 600 nm) as the standard [47].

#### 2.4.3 Photoluminescence Decay Curves

The system is formed by a diode‐pumped solid state mode‐locked Nd:YVO4 laser (Vanguard, Spectra Physics) delivering 2 W of 532 nm light at 76 MHz repetition rate and pulse duration of ∼12 ps that synchronously pumped a cavity‐dumped dye lasers (701‐2, Coherent, delivering 5–6 ps pulses with ∼40 nJ per pulse at 3,4 MHz) working with rhodamine 6G (560‐610 nm) or DCM (620‐700 nm). The laser emission is doubled in BBO crystal to deliver the pulses in 280–305 nm (Rh6G) and 310–350 nm (DCM) spectral range. The output of the Rh6G dye laser was used for excitation (300 nm with a pulse repetition rate of 152 kHz). Intensity luminescence decay curves were made with the emission polarizer set at the magic angle relative to the vertical polarized excitation light. Emission light was passed through a depolarizer before reaching the Jobin‐Yvon iHR320 monochromator with a grating of 100 lines/mm, which selected the light from the samples with a bandwidth of 15–20 nm. The fluorescence was detected by B&H PMC‐100‐02 photomultiplier in photon counting mode. The pulses from PMC‐100‐02 were recorded by the multiscaler MSA 300 from B&H with 5 ns resolution. No less than 20 000 counts were accumulated at the maximum channel.

#### 2.4.4 Transmission Electron Microscopy (TEM)

TEM (80 kV accelerating voltage) images were recorded on a CM120 Philips transmission electron microscope. For TEM sample preparation, a small drop of sample (ca. 5 µL) in water was pipetted onto a carbon film coated on a copper grid. The excess of sample was removed by quick blotting with filter paper leaving a thin spanned film of the sample in the grid, and the grid dried at room temperature.

#### 2.4.5 High Resolution TEM

The HR TEM apparatus used to characterize the AuNCs was a JEOL2100F TEM. Operating at the accelerating voltage of 200 kV. The samples, without dilution, were deposited onto an ultrathin carbon film coated on copper grid (ref.CF400‐CU‐UL, EMS). The excess solution was carefully blotted off using filter paper and air‐dried at room temperature.

## 3 Results and Discussion

Gold nanoclusters (AuNCs) with emission in the near infrared (NIR) were prepared using thiol‐terminated copolymer macro‐ligands, which were synthesized by reversible addition fragmentation chain transfer polymerization (RAFT) controlled radical polymerization. The chain‐end dithiobenzoate group resulting from the RAFT chain transfer agent (CTA) used in the polymerization was converted to a thiol group by aminolysis. This thiol group binds to gold atoms during the AuNCs preparation, and the copolymers are used as stabilizing ligands.

### 3.1 Synthesis of Thiol‐Terminated Copolymer Ligands

The thiol‐terminated polymer macro‐ligands with a molecular weight around 10 000 g.mol^−1^ were synthesized via RAFT polymerization, using tert‐butyl dithiobenzoate (tBDB) as CTA. An homopolymer of PNAM (with no electrostatic charges) was first synthesized in dioxane and characterized as previously optimized [43]. After purification, ^1^H NMR confirmed the structure of the expected polymer (PNAM‐DB) with α‐tert‐butyl (δ = 0.9 ppm) and a ω‐dithiobenzoate (δ = 7–8 ppm) chain‐ends. By comparing the integrals of the peaks corresponding to the monomer units and the chain‐ends, the number‐average molecular weight of the polymer chains was calculated as *M*_n_ = 10 000 g.mol^−1^. This value is in good agreement to the one obtained by SEC‐MALLS (*M*_n_ = 11 300 g.mol^−1^, *Đ* = 1.04) and the expected molecular weight (9 800 g.mol^−1^), calculated from the RAFT initial reactant concentrations and monomer conversion (78% as determined by ^1^H NMR from the raw polymerization sample, Table 1), confirming the very good control of the polymerization.

**TABLE 1:** Individual monomer conversion, number average molecular weight (*M*_n_) and average number of charges per chain of the purified polymers determined by ^1^H NMR.

Polymer	Monomer conversion (%)^a^		Calc. *M*_n_ ^b^ (g mol^−1^)	*M*_n_ (g.mol^−1^)^c^ ^1^H NMR	*n*_charges_ ^d^
	NAM	Co‐monomer			
PNAM‐DB	78	—	9 800	10 000	0
P(NAM_75%_‐*stat*‐AAPTA_25%_)‐DB	75	73	8 300	7 700	12
P(NAM_50%_‐*stat*‐AAPTA_50%_)‐DB	82	86	10 000	9 500	26
P(NAM_75%_‐*stat*‐AMPS_25%_)‐DB	85	86	10 700	9 400	14

^a^ Individual monomer conversion determined by ^1^H NMR using trioxane as internal reference, then used to determine the overall monomer conversion and the final copolymer composition.

^b^ $Calc.\;\;M_{n}=\frac{{\left[NAM\right]}_{0}\times M_{NAM}\times conversion+{\left[comonomer\right]}_{0}\times M_{comonomer}\times conversion}{{\left[CTA\right]}_{0}}+M_{CTA}$.

^c^ *M*_n_ values were determined from the ^1^H NMR spectra of the purified polymers (see Figure S1), by comparing the integrals of the peaks corresponding to the monomer units and the tert‐butyl α‐chain‐end (δ = 0.9 ppm).

^d^ Average number of charges per polymer chain calculated from the individual co‐monomer conversion and *M*_n_ values determined by ^1^H NMR.

To synthesize the positively and negatively charged statistical copolymer ligands, a similar procedure (4 h, 80°C) was followed, copolymerizing NAM with AAPTA and AMPS, respectively. These copolymerization experiments were conducted in dioxane/water mixtures to ensure full solubility of both comonomers and copolymers [48]. In each case, two initial compositions, representing 25 mol% and 50 mol% of the charged monomer compared to NAM, were considered to vary the charge density of the polymers. Individual monomer conversions were followed by ^1^H NMR using trioxane as internal reference. These were then used to determine the average composition of each copolymer (Table 1). After purification by dialysis, the copolymers were characterized by ^1^H NMR in D_2_O (Figure S1). Like for PNAM, the α‐tert‐butyl (δ = 0.9 ppm) and a ω‐dithiobenzoate (δ = 7–8 ppm) chain‐ends were clearly observed and enabled to determine *M*_n_ values in good agreement with those calculated from monomer conversions (Table 1). Aqueous SEC could not be used to analyze the polyelectrolytes due to adsorption onto the columns [49].

To be used as efficient macro‐ligands for the gold nanostructures, the ω‐chain‐end of the polymers was converted into a thiol. Whereas this modification is commonly conducted by aminolysis in organic solvent for PNAM [43, 50], for the charged copolymers it was performed in aqueous medium by reduction with a soluble phosphine (TCEP‐HCl). In all cases, the efficiency of the process was confirmed after purification by ^1^H NMR, showing the complete disappearance of the dithiobenzoate ω‐chain‐end peaks, δ = 7–8 ppm (Figure S1). In addition, the polymers changed from the characteristic pink/salmon color due to the dithiobenzoate ω‐chain‐end, to completely colorless with the thiol ω‐chain‐end.

### 3.2 Synthesis of Chain‐End Grafted Gold Nanoclusters

As previously shown, thiol‐terminated PNAM‐SH can be used as macro‐ligand for the synthesis of highly stable gold nanoclusters (AuNCs) [22]. With this type of water‐soluble macro‐ligand, the synthesis of AuNCs is conducted directly in water at room temperature, a more sustainable route that does not require the use of organic solvents with a suitable phase transfer agent. The macro‐ligand is added to the gold salt before reduction by NaBH_4_ in basic conditions. This process led to PNAM chain‐end stabilized nanoclusters with an Au core of about 1.5 nm as determined by HR‐TEM, and an overall average hydrodynamic diameter of about 15 nm, as measured by an original methodology based of ^1^H DOSY NMR (Table 2). The synthesis of the gold nanoclusters with charged copolymer macro‐ligands followed the same general protocol, since the charged copolymers are also highly water‐soluble. However, the protocol had to be adapted because the electrostatic charges interfere with the nanocluster formation process.

**TABLE 2:** Size (HR TEM and ^1^H DOSY NMR) and linear optical properties of polymer stabilized AuNCs: Emission quantum yield Φ and average lifetime <τ_av_>, maximum intensity excitation and emission wavelengths, λ_exc_ and λ_em_.

Synthesis	*d*_Au core_	*D*_h_	Linear optical properties			
	HR TEM (nm)	^1^H DOSY NMR (nm)	*ϕ* (%)	*λ*_exc max_ ^a^ (nm)	*λ*_em max_ ^b^ (nm)	*<τ_av_>* (µs)
MHA‐AuNCs	1.9 ± 0.4	3.7 ± 0.6	0.05	350	∼800	0.4
PNAM‐AuNCs	1.5 ± 0.3	15 ± 1	12	364	711	2.2
P(NAM_75%_‐*stat*‐AAPTA_25%_)‐AuNCs	1.6 ± 0.3	11 ± 1	8	285	675	2.1
P(NAM_50%_‐*stat*‐AAPTA_50%_)‐AuNCs	1.6 ± 0.5	10 ± 1	8	285	705	0.9
P(NAM_75%_‐*stat*‐AMPS_25%_)‐AuNCs	5 ± 1^c^	—	—	300^c^	665^c^	2.1^c^

^a^ λ_em_ = 720 nm.

^b^ λ_exc_ = 320 nm.

^c^ Mixture of gold nanoparticles and gold nanoclusters.

Indeed, using the P(NAM‐*stat*‐AAPTA)‐SH macro‐ligands, regardless of the NAM/AAPTA molar ratio, the AuNCs synthesis showed some visual differences compared to that using PNAM‐SH. The mixing of the yellow‐colored HAuCl_4_ and colorless P(NAM‐*stat*‐AAPTA)‐SH aqueous solutions led to the precipitation of the HAuCl_4_ salt (ionic complexation of AuCl_4_^−^ salt by the cationic polymer) [36]. The precipitate then slowly re‐solubilized under stirring, and more rapidly after the addition of NaOH. This behavior reflects the progressive reduction of Au(III) to Au(I) and the formation of soluble Au(I)‐S‐polymer complexes, thanks to the thiol chain‐end of the polymer that strongly interact with gold. NaOH has an essential role in controlling the formation of the cationic AuNCs: first in the formation of the Au(I)‐S‐polymer complex, and second in decreasing the reducing power of NaBH_4_ [36]. The dispersion then turned to light brown, and progressively darkened with the addition of NaBH_4_ reducing solution, as expected. The preparation of AuNCs by this method did not lead to the formation of larger gold nanoparticles (AuNPs) over a wide range of NaBH_4_/Au ratios from 1.73 to 9.43 for P(NAM_75%_‐*stat*‐AAPTA_25%_). In addition, the cationic P(NAM‐*stat*‐AAPTA)‐AuNCs exhibited excellent colloidal stability, noticeably, even after the addition of reducing agents that commonly destabilize the other AuNCs. This may be due to the quaternary amine groups in the polymer, since amines can also stabilize the AuNCs, as well as the hydrophobic contributions of AAPTA.

The preparation of AuNCs in the presence of the anionic P(NAM‐*stat*‐AMPS) macro‐ligands in similar conditions also led to visual differences relatively to the synthesis of PNAM‐AuNCs, but in this case only when adding the NaBH_4_ reduction agent. The dispersion turned more reddish, compared to the classical brown color of AuNCs dispersions. This red‐color is usually an indication of the formation of larger gold nanoparticles (AuNPs, diameters above 2–3 nm) [51]. The change of synthesis parameters, such as the Au salt / polymer / NaBH_4_ ratios, did not prevent the formation of the larger AuNPs. A mixture of both AuNCs and AuNPs was then formed, with the dispersion presenting more or less red/brown color. Only the addition of more NaBH_4_, up to 5 times the initial quantity, seemed to reduce the proportion of AuNPs to some extent. These observations for the polyanionic P(NAM‐*stat*‐AMPS)‐SH ligands were not reported for the synthesis of AuNCs with small anionic ligands possessing only one negative charge per ligand [32, 52]. This may indicate that the polyanionic P(NAM‐*stat*‐AMPS)‐SH ligand is either hindering the stabilization of the AuNCs or the reduction power of NaBH_4_, both factors being known to favor the formation of larger gold nanoparticles.

### 3.3 Size Characterization of the Polymer‐Gold Nanohybrids

High‐resolution transmission electron microscopy (HR‐TEM) was used to image the gold core of the nanostructures, that appeared spherical in all cases, and to determine its size distribution. The high voltage applied did not allow the observation of the (mostly) electron‐transparent polymer grafted to the core.

Very similar AuNCs were obtained both with PNAM‐SH and cationic P(NAM‐*stat*‐AAPTA)‐SH macro‐ligands, independently of the AAPTA content. P(NAM_75%_‐*stat*‐AAPTA_25%_)‐AuNCs and P(NAM_50%_‐*stat*‐AAPTA_50%_)‐AuNCs (Figure 2A,B), exhibited a gold core size distribution and an average diameter of 1.6 nm (Figure 2D), very close to those of PNAM‐AuNCs (average diameter of 1.5 nm, Table 1). In contrast, HR TEM images of the sample obtained with anionic P(NAM‐*stat*‐AMPS)‐SH macro‐ligand (Figure 2C), confirmed the formation of larger nanoparticles (AuNPs, d > 2 nm, higher contrast), in addition to the AuNCs. Analysis of the images revealed a broad size distribution (2 to 8 nm), characterized by an average Au core diameter around 5 nm (Figure 2D). Conventional transmission electron microscopy (TEM) (Figure S2) revealed a random dispersion of the AuNPs on the grid, without pattern, while the AuNCs were not visualized due to the lower resolution. This indicates that polymer‐stabilized gold nanoparticles were formed in this case, rather than large polymer assemblies containing multiple gold nanoparticles.

**FIGURE 2:**
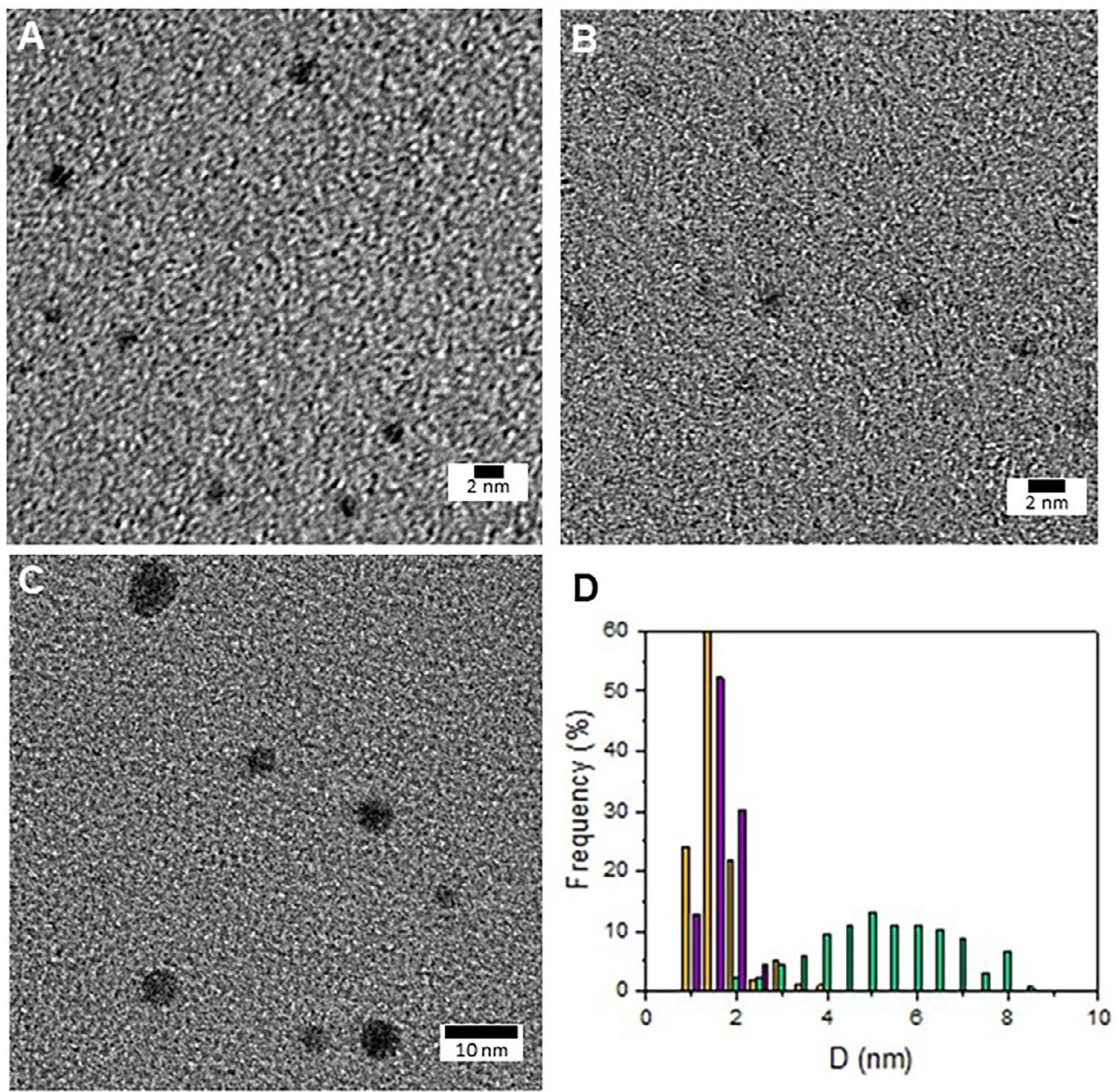
HR‐TEM images of (A) P(NAM_75%_‐*stat*‐AAPTA_25%_)‐AuNCs (scale bar 2 nm), (B) P(NAM_50%_‐*stat*‐AAPTA_50%_)‐AuNCs (scale bar 2 nm), and (C) P(NAM_75%_‐*stat*‐AMPS_25%_)‐AuNPs (scale: 10 nm). (D) HR TEM size distribution of the gold core of the corresponding samples (purple: P(NAM_75%_‐*stat*‐AAPTA_25%_); orange: P(NAM_50%_‐*stat*‐AAPTA_50%_); green: P(NAM_75%_‐*stat*‐AMPS_25%_)‐AuNPs).

The average hydrodynamic diameter (*D*_h_) of the AuNCs in dispersion was analyzed by the ^1^H DOSY NMR method (Figure 3), that we previously developed and validated for PNAM‐AuNCs [22]. The average *D*_h_ values of 11 nm for P(NAM_75%_‐*stat*‐AAPTA_25%_)‐AuNCs and 10 nm for P(NAM_50%_‐*stat*‐AAPTA_50%_)‐AuNCs dispersed in D_2_O (Table S1), were slightly lower than the one obtained for PNAM‐AuNCs (*D*_h_ = 15 nm). This may indicate that the hydrophobic contribution of the AAPTA units favors a less expanded conformation of the copolymer chains at the gold core surface, despite the repulsion between the cationic charges. For the sample prepared with the anionic P(NAM_75%_‐*stat*‐AMPS_25%_)‐SH macro‐ligand, it was not possible to determine an average *D*_h_ by the same DOSY NMR analysis, as the signal attenuation was not sufficient with the gradient strength provided by the probe. This was additional evidence that larger AuNPs were formed in that case.

**FIGURE 3:**
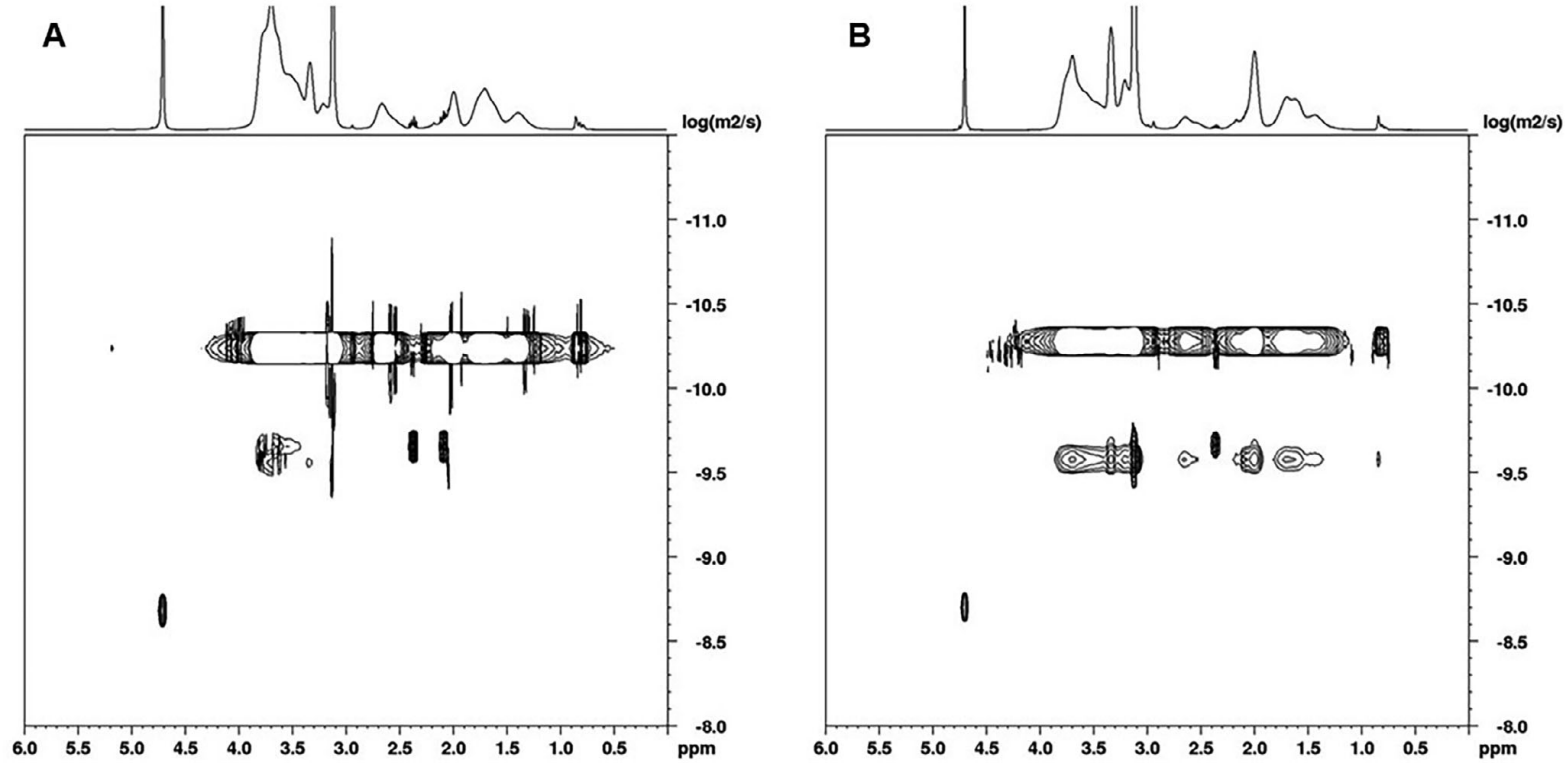
^1^H DOSY NMR of (A) P(NAM_75%_‐*stat*‐AAPTA_25%_)‐AuNCs dispersion in D_2_O. (B) P(NAM_50%_‐*stat*‐AAPTA_50%_)‐AuNCs dispersion in D_2_O.

Finally, as previously observed for the PNAM‐AuNCs, the AuNCs synthesized with the RAFT polyelectrolytes showed a very high stability upon storage (months).

### 3.4 Optical Properties of the Polymer‐Gold Nanohybrids

The optical properties of the polymer‐gold nanohybrids were studied in terms of UV–vis absorption, photoluminescence spectra, photoluminescence quantum yield and emission decay curves.

The cationic clusters, P(NAM_75%_‐*stat*‐AAPTA_25%_)‐AuNCs and P(NAM_50%_‐*stat*‐AAPTA_50%_)‐AuNCs (Figure 4A,B), exhibited the same advantages than PNAM‐stabilized AuNCs (Table 2; Figure S4), in particular, the absence of surface plasmon resonance (SPR) band around 520 nm that confirmed the formation of only AuNCs (*d* < 2 nm). In addition, the photoluminescence emission spectrum of P(NAM‐*stat*‐AAPTA)‐AuNCs, for both compositions, showed NIR emission (*λ*_em max_ ≈ 700 nm) with a large Stokes shift (between 390 and 420 nm), showing no overlap between the emission and the excitation spectra. This was consistent with the formation of the most common and stable Au_25_ AuNCs with 18 ligands per cluster [22].

**FIGURE 4:**
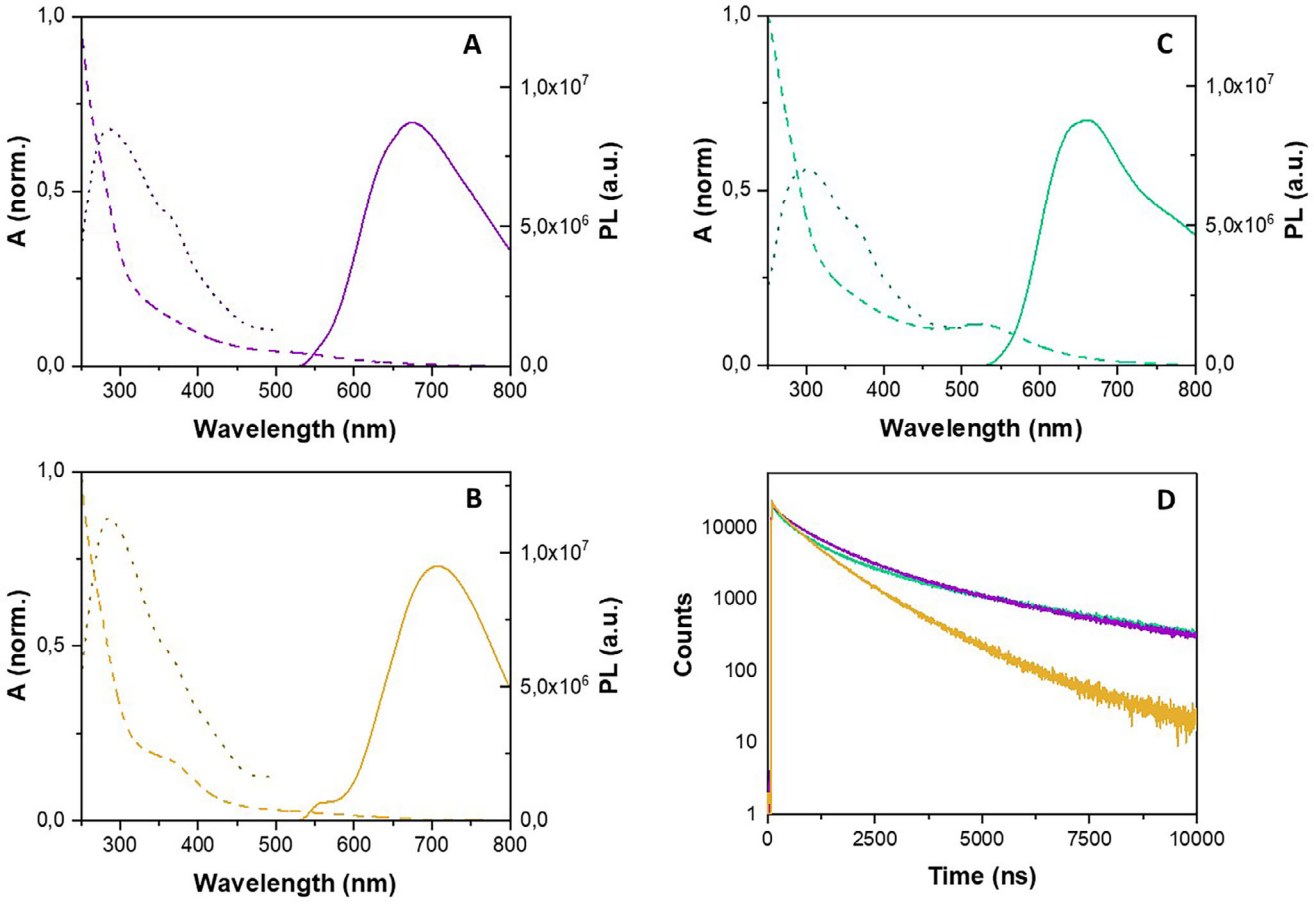
UV–vis absorption (dashed line), photoluminescence excitation (dotted line, λ_em_ = 720 nm) and photoluminescence emission (full line, λ_exc_ = 320 nm) spectra of P(NAM_75%_‐stat‐AAPTA_25%_)‐AuNCs (A), P(NAM_50%_‐*stat*‐AAPTA_50%_)‐AuNCs (B) and P(NAM_75%_‐*stat*‐AMPS_25%_)‐AuNCs (C). Photoluminescence decays (D) for polymer‐stabilized AuNCs by excitation at 300 nm and emission at 750 nm (purple: P(NAM_75%_‐*stat*‐AAPTA_25%_)‐AuNCs); yellow: P(NAM_50%_‐*stat*‐AAPTA_50%_)‐AuNCs); green: P(NAM_75%_‐*stat*‐AMPS_25%_)‐AuNCs).

The cationic quaternary amine group and/or the hydrophobic contribution of the AAPTA units led to several noticeable differences for the P(NAM‐*stat*‐AAPTA)‐AuNCs compared to PNAM‐AuNCs. First, both the P(NAM_75%_‐*stat*‐AAPTA_25%_)‐AuNCs and P(NAM_50%_‐*stat*‐AAPTA_50%_)‐AuNCs presented a small distinctive absorption band around 350 nm, which can be attributed to the n‐π* transition of the amine group, since its intensity increased with the increase of AAPTA in the copolymer [53, 54]. Second, the NIR photoluminescence of P(NAM‐*stat*‐AAPTA)‐AuNCs was slightly blue‐shifted compared to PNAM‐AuNCs, although the effect was smaller for the larger AAPTA content of the copolymer. This may be attributed to the change in the electronic environment of the AuNCs, due to the amine groups and the charge. Third, the P(NAM‐*stat*‐AAPTA)‐AuNCs exhibited a photoluminescence quantum yield, *Φ =* 8%, that did not change with the NAM/AAPTA molar ratio (Table 2) but was lower than for the PNAM‐AuNCs (*Φ =* 12%). Finally, the number‐averaged emission lifetime of P(NAM_75%_‐*stat*‐AAPTA_25%_)‐AuNCs (<*τ*>  = 2.1 µs) was similar to the one of PNAM‐AuNCs (<*τ*> = 2.2 µs), but decreased for P(NAM_50%_‐*stat*‐AAPTA_50%_)‐AuNCs (<*τ*> = 0.9 µs), probably because the excess charge affects the radiative rate constant (Table S2). The effect of the copolymer composition on photoluminescence decay kinetics (Figure 4D) is probably related to electrostatic interactions that can increase the exposure of the AuNCs core to the aqueous environment (relative to PNAM‐AuNCs), increasing emission quenching. Nevertheless, the average photoluminescence lifetime of the polycationic AuNCs are still much higher than those of AuNCs stabilized by small molecular ligands.

The synthesis of AuNCs using P(NAM‐*stat*‐AAPTA)‐SH polycationic macro‐ligands thus successfully yielded NIR‐emitting AuNCs with no major changes in the size and optical properties compared to PNAM‐AuNCs.

For the samples prepared with the anionic P(NAM‐*stat*‐AMPS)‐SH macro‐ligand, the SPR band at 520 nm in the UV‐vis absorption spectrum (Figure 4C) was a clear indication of the formation of larger AuNPs (d > 2 nm). Nonetheless, a broad photoluminescence emission band in the NIR (between 700 and 800 nm) was still observed, regardless of the amount of NaBH_4_ added during the synthesis. This demonstrated that the sample was composed by both NIR‐emitting AuNCs and non‐emissive plasmonic AuNPs. The photoluminescence decay curve was similar to those of P(NAM_75%_‐*stat*‐AAPTA_25%_)‐AuNCs (Table 2), showing that the emissive AuNCs formed in the presence of P(NAM_75%_‐*stat*‐AMPS_25%_)‐SH polymer had similar optical properties to those of P(NAM_75%_‐*stat*‐AAPTA_25%_)‐AuNCs. As noted above, the formation of AuNPs likely reflects that the P(NAM‐*stat*‐AMPS)‐SH polyanion macroligand is attenuating the reduction strength of NaBH_4_, and/or is lowering the efficiency of coordination of the copolymer to the Au atoms, and thus the stabilization of the AuNCs. The decrease in the coordination efficiency of the copolymer to the Au atoms may be related to the electrostatic interaction between the multiple anionic groups in polymer chains and the positive kernel of the AuNCs.

## 4 Conclusions

NIR‐emitting gold nanoclusters (AuNCs) were prepared using thiol‐terminated polycationic and polyanionic copolymer macro‐ligands obtained by RAFT polymerization. In addition to their thiol ω‐chain‐end, both polyelectrolytes bear permanent electrostatic charges (quaternary amines and sulfonate, respectively), and their charge density was varied. While the negatively charged P(NAM‐*stat*‐AMPS)‐SH, yielded a mixture of AuNCs and larger AuNPs (*d* > 2 nm), the positively charged P(NAM‐*stat*‐AAPTA)‐SH led to highly stable NIR‐emitting AuNCs, with core diameter *d* < 2 nm, and hydrodynamic diameter *D*_h_ ∼10 nm.

Our AuNCs, stabilized with end‐grafted water‐soluble polymers synthesized by RAFT polymerization, offer a good alternative to protein and dendrimer protected AuNCs, featuring NIR emission with large Stokes shifts, and large photoluminescence quantum yield and lifetimes. The synthetic route is simple and water‐based, with potentially scalable production. In addition, we demonstrate that our novel approach can be used to obtain (multi‐)functionalized AuNCs. The polycationic AuNCs may find applications, for example in the biomedical field, as gene delivery vectors, antimicrobials or bioimaging probes. It can also be a useful building block for the development of advanced self‐assembled structures.

## Funding

Fundação para a Ciência e a Tecnologia (FCT‐Portugal) and COMPETE (FEDER) within projects PTDC/CTM‐COM/1581/2021, UID/00100/2025, UID/PRR/00100/2025, and LA/P/0056/2020. BC FCT PhD grant PD/BD/137511/2018. French‐Portuguese Hubert Curien Partnership “PESSOA” Program n 44648XM.

## Conflicts of Interest

The authors declare no conflicts of interest.

## Supporting information




**Supporting File**: marc70278‐sup‐0001‐SuppMat.pdf.

## Data Availability

The data that support the findings of this study are available from the corresponding author upon reasonable request.
