# Supplementary material for: Chain‐End Grafting of RAFT Polyelectrolyte Macroligands to Stabilize Near‐Infrared‐Emitting Gold Nanoclusters
Source: Macromol Rapid Commun. 2026 Apr 9;47(19):e00007. doi: 10.1002/marc.202600007 (PMC13639197; doi:10.1002/marc.202600007)
Supplement: Supplementary file 1 [file MARC-47-e00007-s001.pdf]

Chain-end grafting of RAFT polyelectrolyte macroligands to stabilize near-infrared-emitting gold nanoclusters

Bárbara Casteleiro,[a,b] Pierre Alcouffe,[b] Fernande Da Cruz,[b] Agnès Crépet,[b] José Manuel Gaspar Martinho,[a] Marie-Thérèse Charreyre,[b] José Paulo Sequeira Farinha,[a] Arnaud Favier[b]

[a]Centro de Química Estrutural, Institute of Molecular Sciences and Department of Chemical Engineering, Instituto Superior Técnico, University of Lisbon, 1049-001 Lisbon, Portugal

[b] Univ Lyon, Université Lyon 1, INSA de Lyon, CNRS, Laboratoire Ingénierie des Matériaux Polymères, UMR5223, F-69621 Villeurbanne, France

Supporting Information

1. Polyelectrolyte characterization.. 2

2. Polymer-stabilized AuNCs characterization 3

1. Charged Polymers Characterization

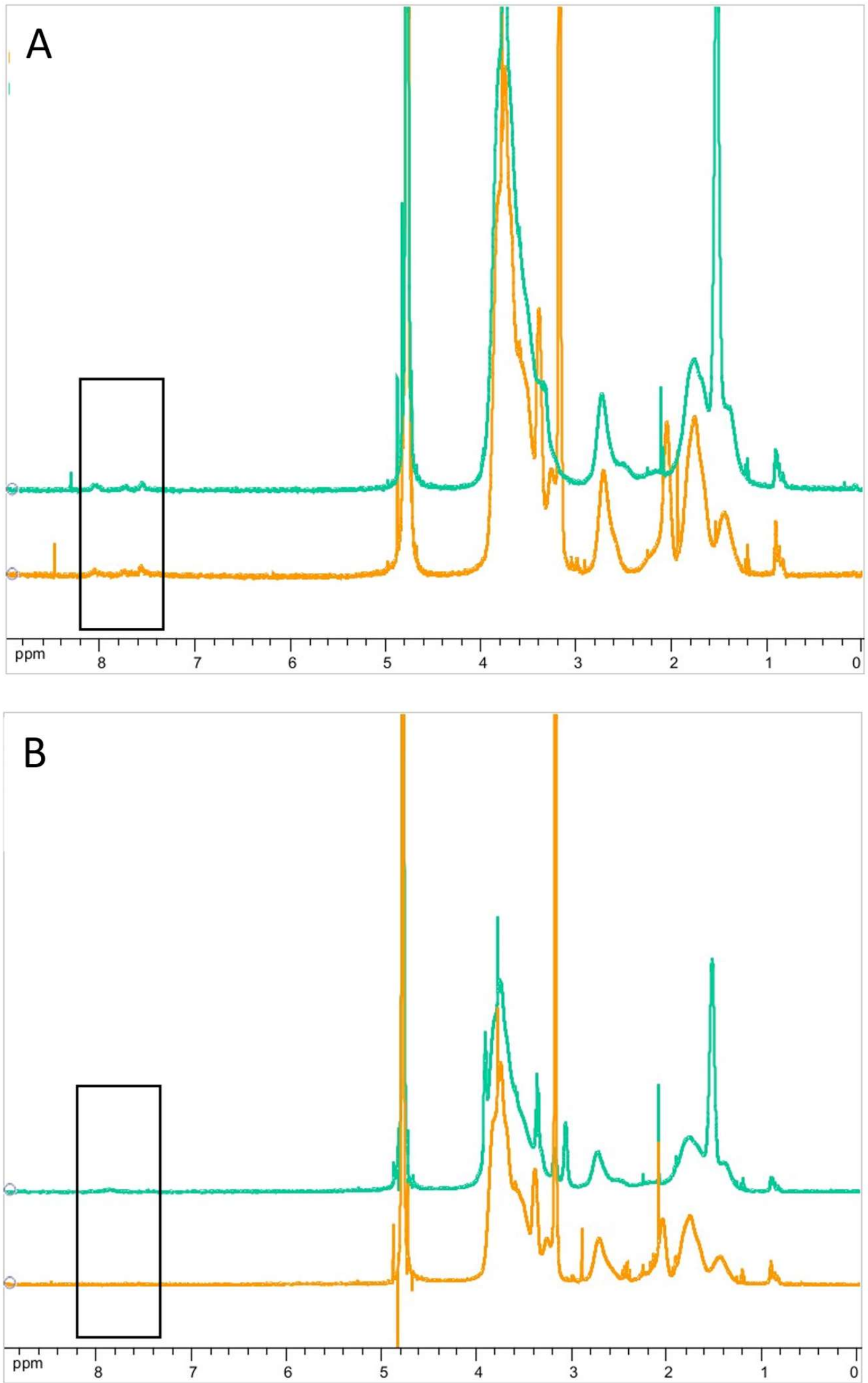

Figure S1. ^1H NMR spectra in D_2O of the purified copolymer samples before (A) and after (B) reduction of their dithiobenzoate (-DB) ω-chain-end into a thiol (-SH) ω-chain-end. P(NAM-stat-AMPS) samples in green, P(NAM-stat-AAPTA) samples in orange). In both cases, full disappearance of the characteristic dithiobenzoate aromatic peaks between 7-8 ppm confirmed the efficient reduction.

2. Polymer-stabilized AuNCs Characterization

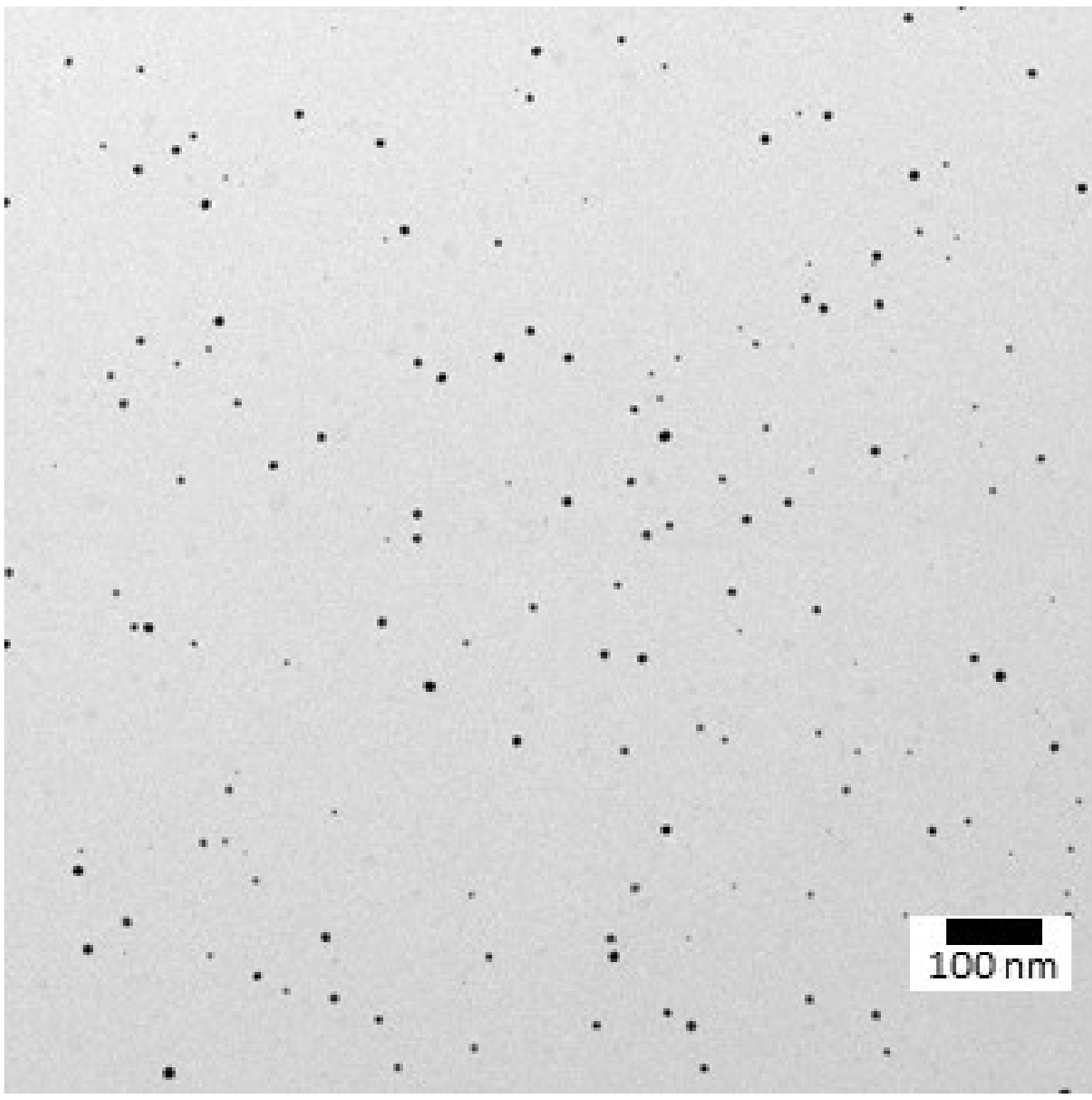

Figure S2. TEM image of P(NAM75%-stat-AMPS25%)-AuNPs (scale: 100 nm).

Table S1. Diffusion coefficients (D) and hydrodynamic diameter determined in D_2O by ^1H NMR DOSY analyses of the polymer-stabilized AuNCs (after ultrafiltration with 3kDa MWCO) and of the corresponding polymers alone.

	Sample	D ($m^2\,s^{-1}$)	D_h (nm)
AuNCs	PNAM-AuNCs	2.7 x10^{-11}	15
	P($NAM_{75\%}$-*stat*-$AAPTA_{25\%}$)-AuNCs	3.9 x10^{-11}	11
	P($NAM_{50\%}$-*stat*-$AAPTA_{50\%}$)-AuNCs	4.2 x10^{-11}	10
Polymers	PNAM	5.5 x10^{-11}	7.6
	P($NAM_{75\%}$-*stat*-$AAPTA_{25\%}$)	6.8 x10^{-11}	6.2
	P($NAM_{50\%}$-*stat*-$AAPTA_{50\%}$)	6.3 x10^{-11}	6.6
	P($NAM_{75\%}$-*stat*-$AMPS_{25\%}$)	6.4 x10^{-11}	6.5

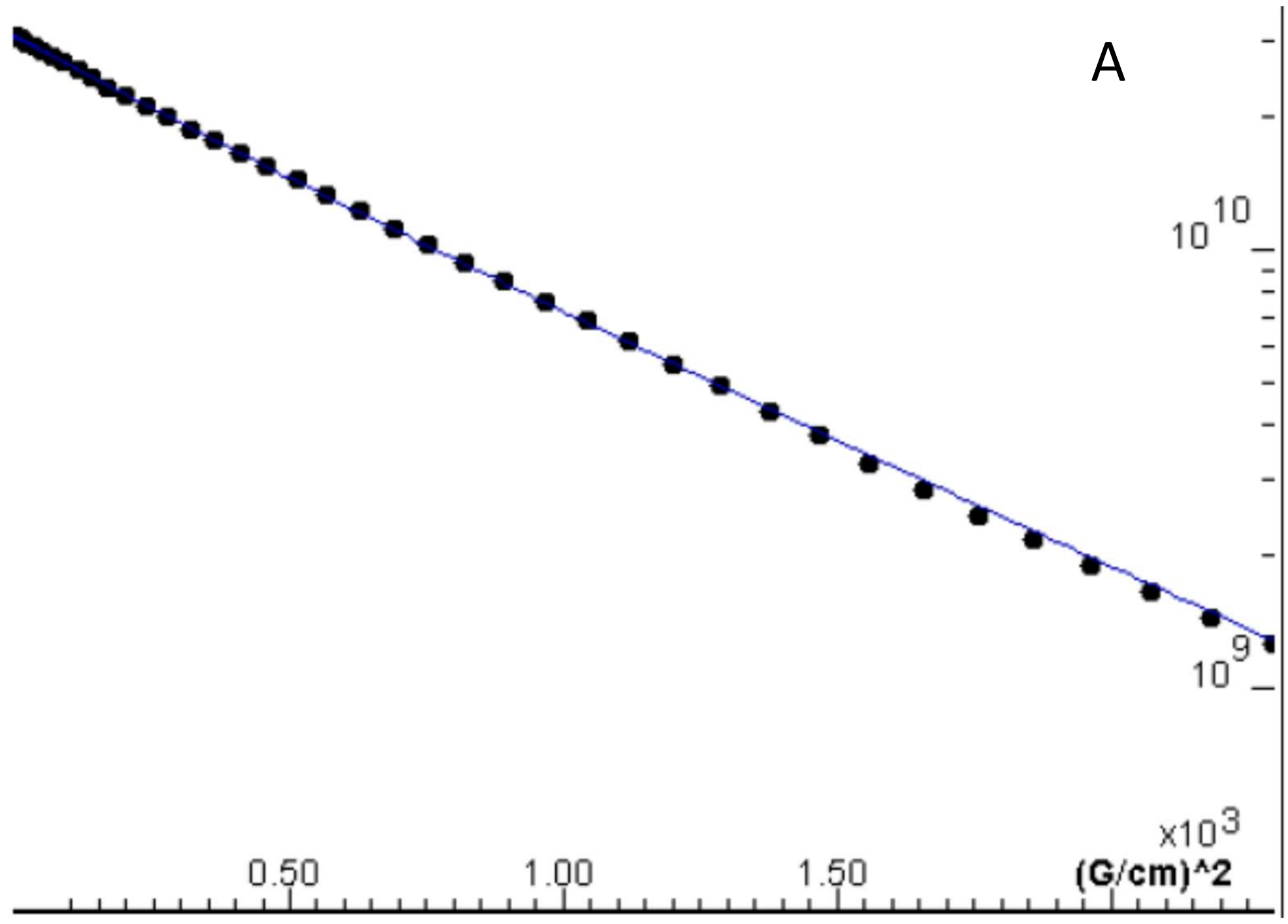
A
10^10
10^9
x10^3
0.50
1.00
1.50
(G/cm)^2

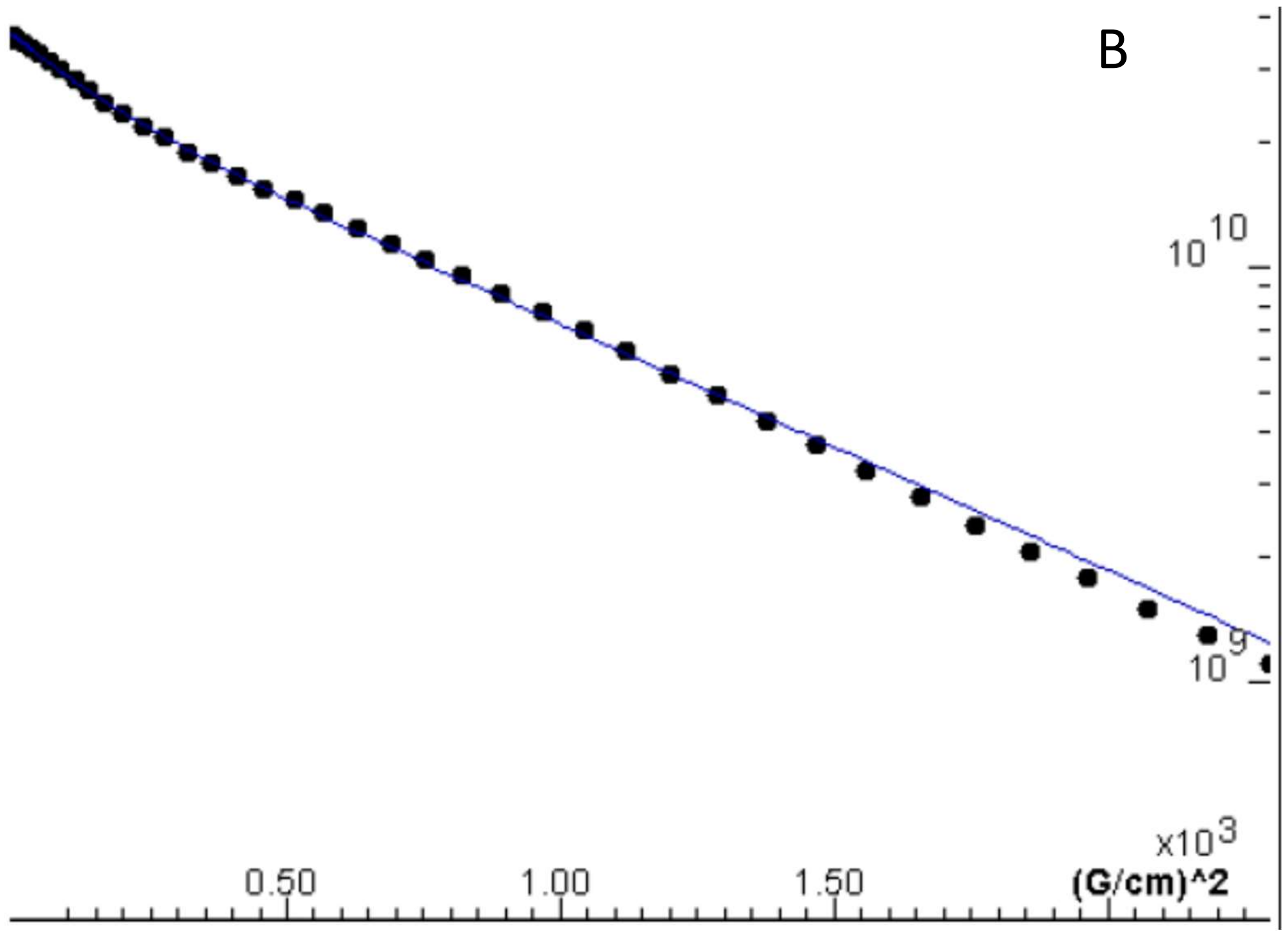
B
10^10
10^9
x10^3
0.50
1.00
1.50
(G/cm)^2

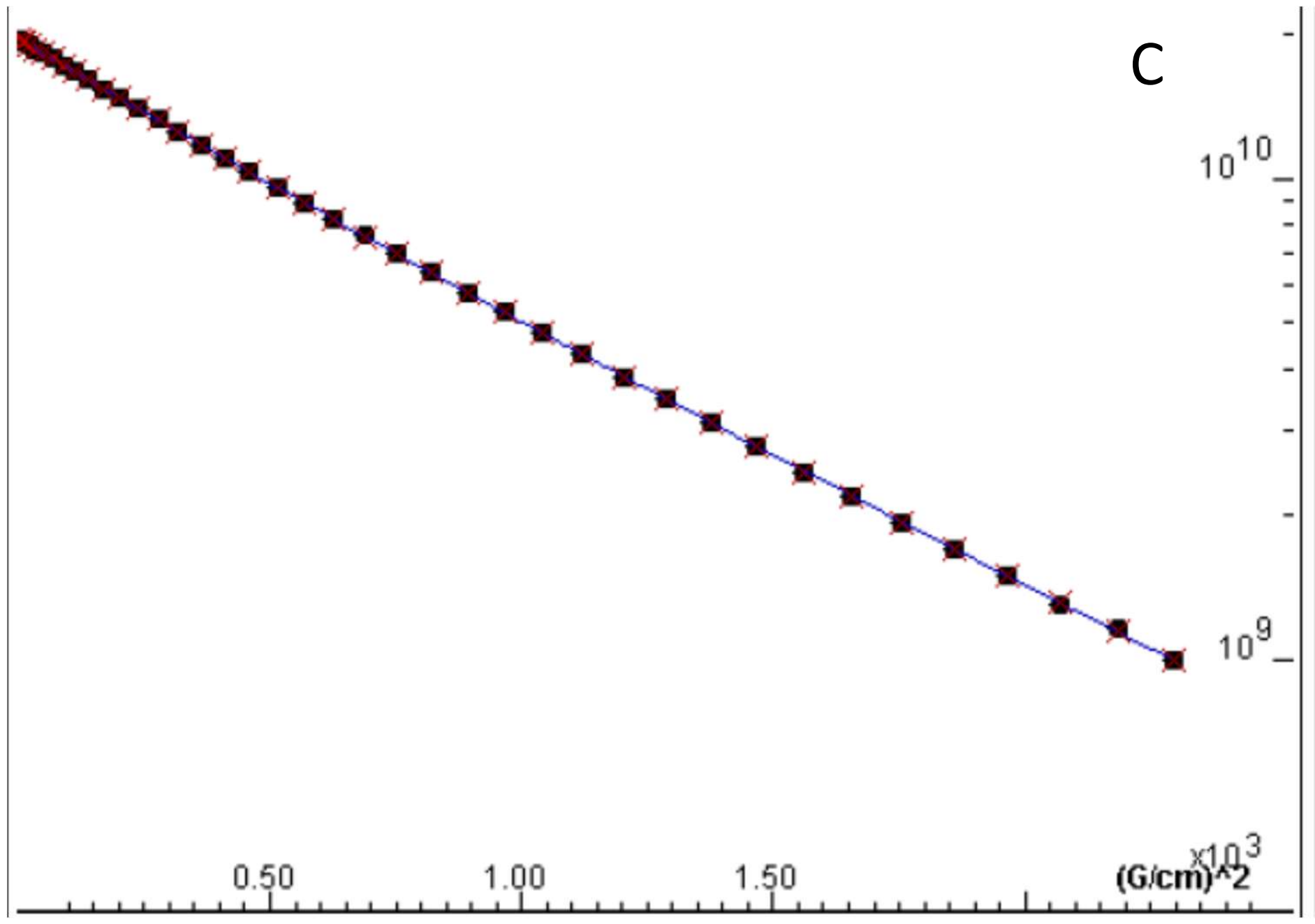
C
10^{10}
10^9
0.50
1.00
1.50
x10^3
(G/cm)^2

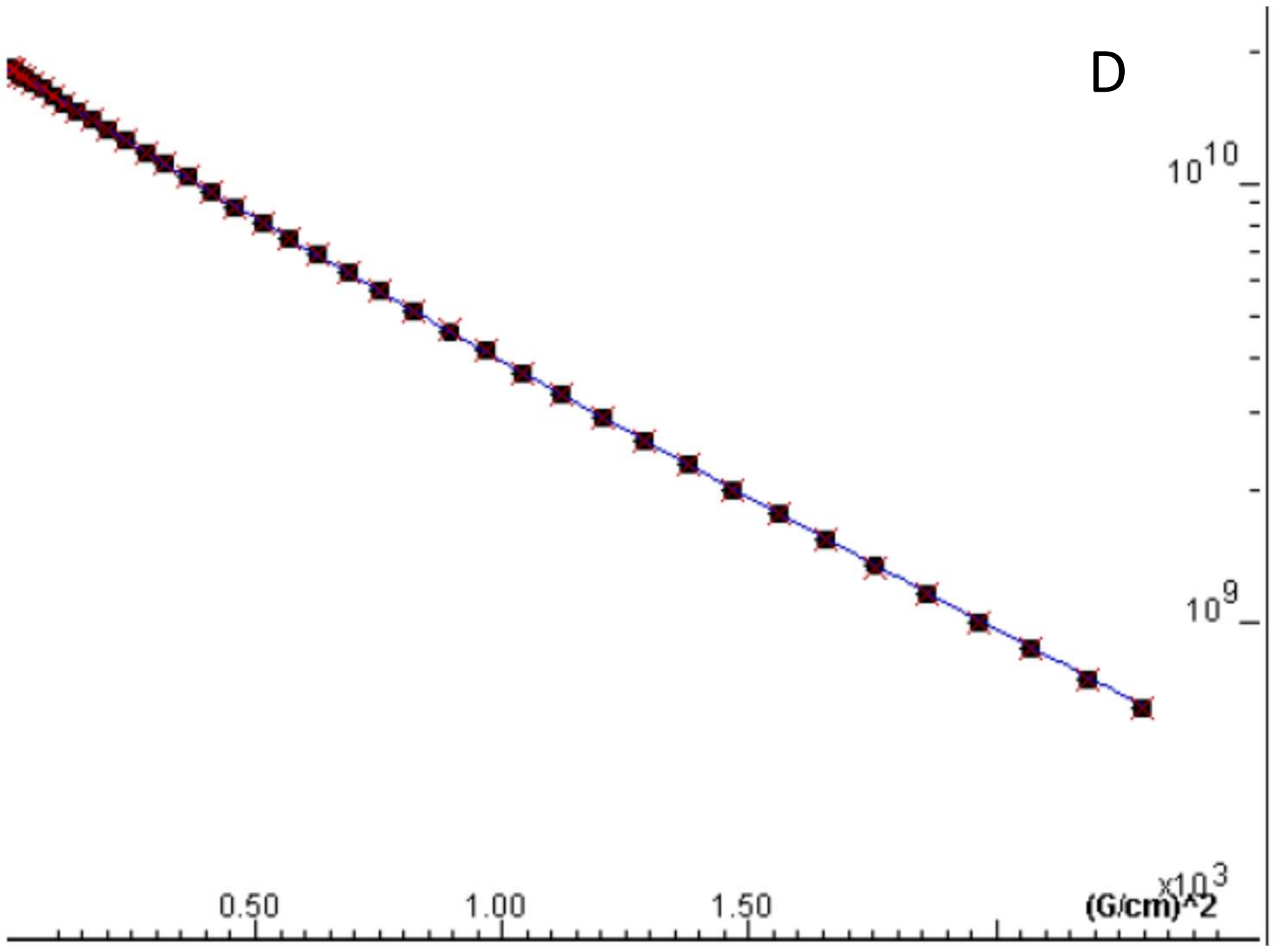
D
10^{10}
10^9
0.50
1.00
1.50
x10^3
(G/cm)^2

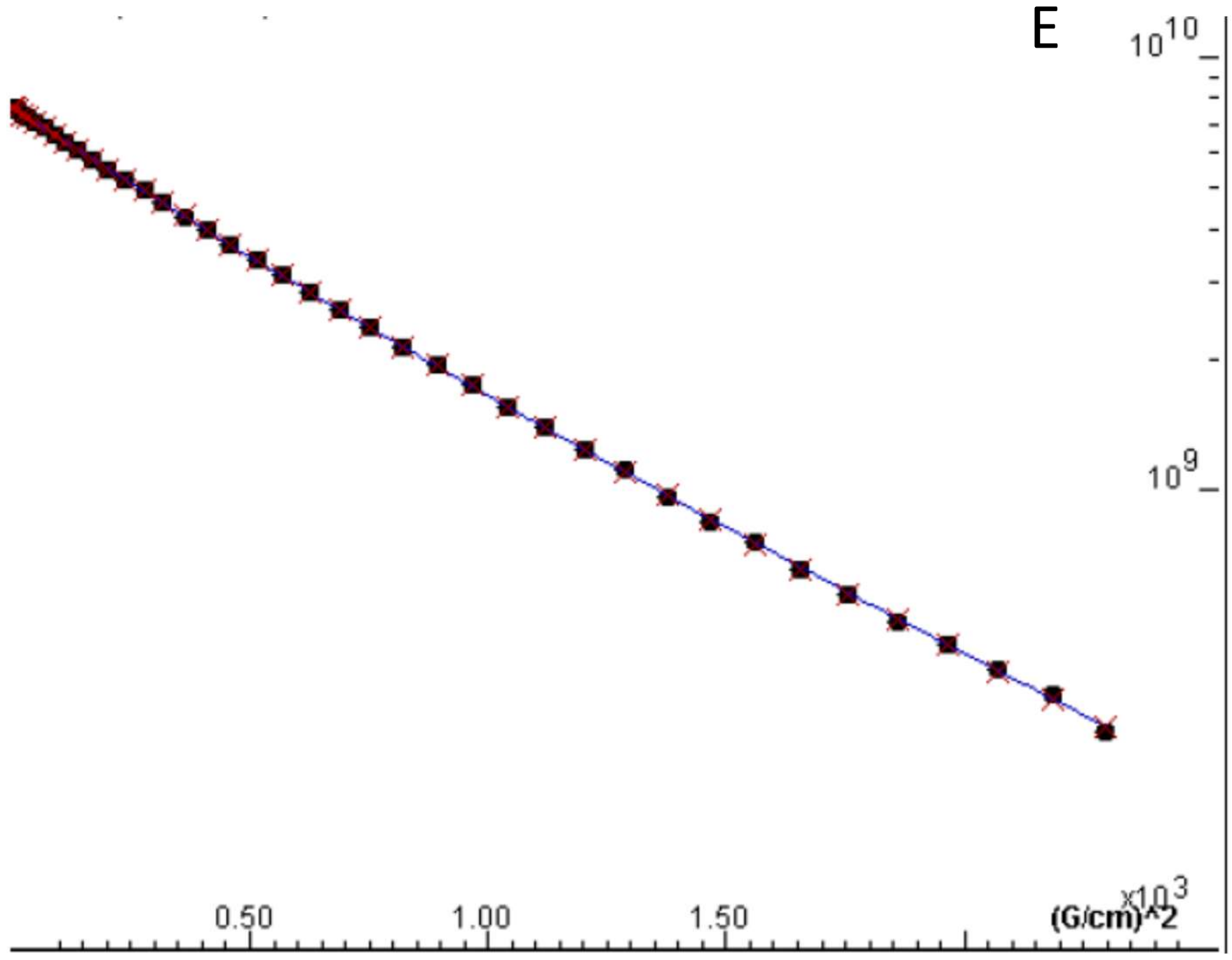

Figure S3. ^{1}H DOSY NMR decays: ^{1}H signal Intensity decays (Log I) as a function of gradient strength (g2) and their corresponding fitting curves for A) P(NAM$_{75\%}$-*stat*-AAPTA$_{25\%}$)-AuNCs, B) P(NAM$_{50\%}$-*stat*-AAPTA$_{50\%}$)-AuNCs and the copolymers alone: C) P(NAM$_{75\%}$-*stat*-AAPTA$_{25\%}$) D) P(NAM$_{50\%}$-*stat*-AAPTA$_{50\%}$) E) P(NAM$_{75\%}$-*stat*-AMPS$_{25\%}$)

Photoluminescence decay curves

Table S2. Photoluminescence decay parameters for the fit of the experimental decay curves with a sum of 3 exponentials, $I(t) = \sum_{i=0}^{3} \alpha_i \times \exp(-t/\tau_i)$. The average decay time was $<\tau> = \sum \alpha_i \tau_i / \sum \alpha_i$. The quality of the fit was judged by the χ^2 values.

	α_1 (%)	τ_1 (ns)	α_2 (%)	τ_2 (ns)	α_3 (%)	τ_3 (ns)	$<\tau>$ (ns)	X^2
PNAM-AuNCs	1.4%	65	7.4%	370	91.2%	2396	2214	1.12
P(NAM$_{75\%}$-*stat*-AAPTA$_{25\%}$)-AuNCs	4.2	190	51.2	1028	44.6	3406	2053	1.09
P(NAM$_{50\%}$-*stat*-AAPTA$_{50\%}$)-AuNCs	15.0	305	84.4	1015	0.6	2704	919	1.40
P(NAM$_{75\%}$-*stat*-AMPS$_{25\%}$)-AuNCs	5.1	172	40.0	819	54.9	3256	2124	1.15

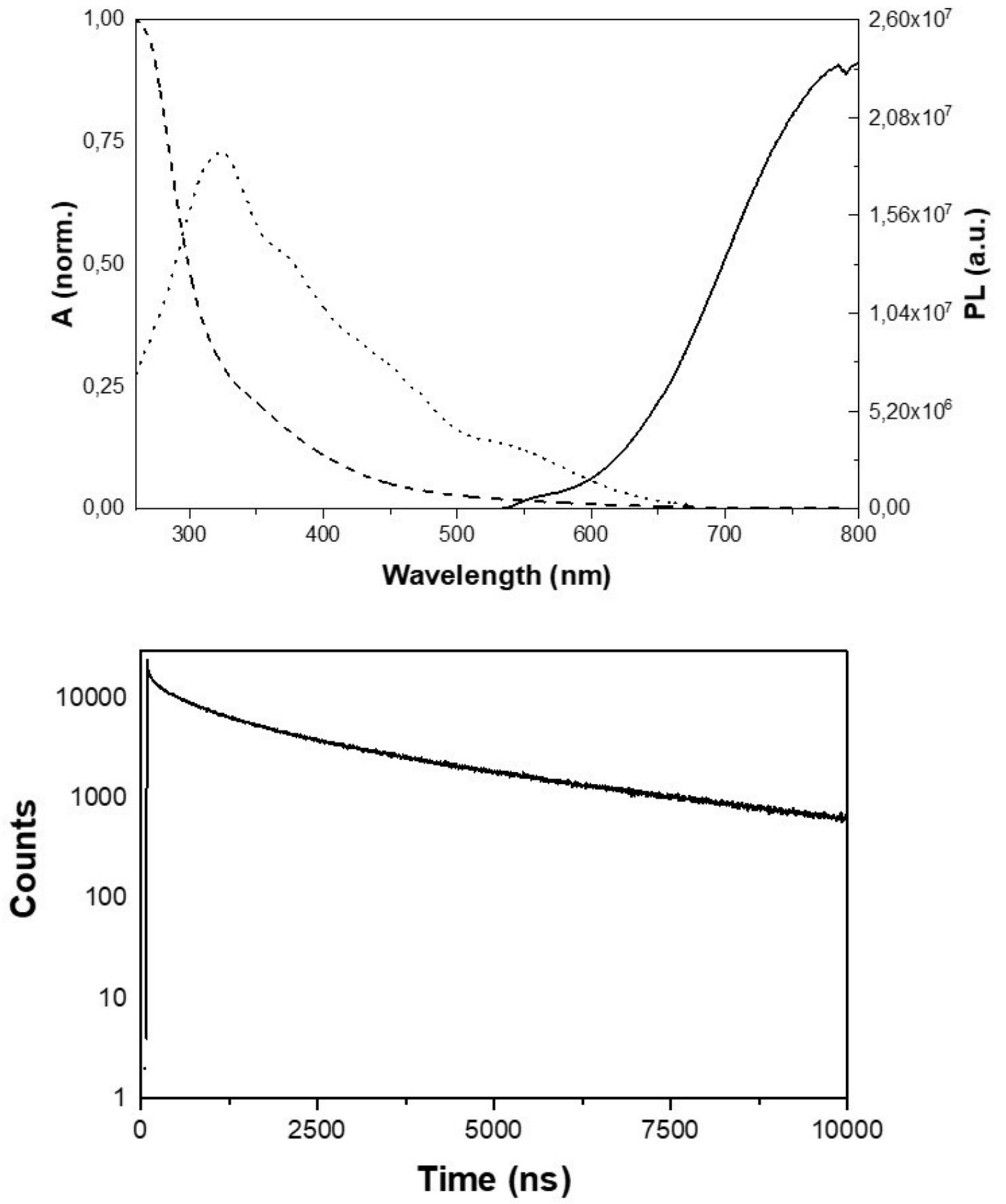

Figure S4. Spectrophysical properties of PNAM-AuNCs: (Top) UV-vis absorption (dashed line), photoluminescence excitation (dotted line, λ_{em}=720 nm) and photoluminescence emission (full line, λ_{exc}=330 nm) spectra; (Bottom) Photoluminescence decay
